# Effect of Green and Red Thai Kratom (*Mitragyna speciosa*) on pancreatic digestive enzymes (alpha-glucosidase and lipase) and acetyl-carboxylase 1 activity: A possible therapeutic target for obesity prevention

**DOI:** 10.1371/journal.pone.0291738

**Published:** 2023-09-21

**Authors:** Atikarn Janthongkaw, Sirinthip Klaophimai, Tanaporn Khampaya, Supaporn Yimthiang, Yilin Yang, Ruixue Ma, Apirak Bumyut, Phisit Pouyfung

**Affiliations:** 1 Environmental, Safety Technology and Health, School of Public Health, Walailak University, Nakhon Si Thammarat, Thailand; 2 Department of Oral Biology, Faculty of Dentistry, Mahidol University, Ratchathevi, Bangkok, Thailand; 3 Occupational Health and Safety, School of Public Health, Walailak University, Nakhon Si Thammarat, Thailand; 4 Department of Internal Medicine, Section of Digestive Diseases, Yale School of Medicine, New Haven, United States of America; 5 Department of Gastroenterology, Xiangya Hospital of Central South University, Changsha, Hunan, China; 6 Environmental Health and Technology, School of Public Health, Walailak University, Nakhon Si Thammarat, Thailand; 7 Biomass and Oil Palm Center of Excellence, Walailak University, Nakhon Si Thammarat, Thailand; Cukurova University: Cukurova Universitesi, TURKEY

## Abstract

Regular use of Thai kratom has been linked to reduced blood triglyceride levels and body mass index (BMI) in healthy individuals. We analyzed Green Thai Kratom (GTK) and Red Thai Kratom (RTK) to investigate their effects on pancreatic digestive enzymes. The ethanol extracts of GTK and RTK inhibited lipase activity more strongly than alpha-glucosidase activity, suggesting the presence of lipase inhibitors. Mitragynine, the major compound in GTK, showed potent lipase inhibition and moderate alpha-glucosidase inhibition. Quercetin, found in both extracts, strongly inhibited alpha-glucosidase but had limited effects on lipase. These findings suggest that mitragynine and quercetin may hinder triglyceride and starch digestion. Combination inhibition studies revealed synergistic effects between mitragynine and quercetin on alpha-glucosidase activity. Additionally, both GTK and RTK extracts reduced fat accumulation in 3T3-L1 adipocyte cells, with quercetin specifically inhibiting Acetyl-CoA carboxylase 1 (ACC1), a key enzyme in fatty acid biosynthesis. Thus, GTK and RTK extracts, particularly mitragynine and quercetin, exhibit potential anti-obesity effects. We report the novel finding that Thai kratom inhibits de novo fatty acid synthesis by targeting ACC1, resulting in decreased fat accumulation in adipocytes. Regular use of Thai kratom in specific populations may improve blood triglyceride levels and reduce BMI by inhibiting lipase, alpha-glucosidase, and ACC1 activity. Further clinical trials are needed to determine optimal dosage, duration, toxicity levels, and potential side effects of Kratom use.

## Introduction

Obesity and obesity-related diseases including cardiovascular disease, non-alcoholic fatty liver disease (NAFLD), and type 2 diabetes are the leading causes of death worldwide [1]. Obesity has become prevalent in all age groups, affecting more than 1.9 billion adults (>18 years old) and over 340 million children and adolescents (5–19 years old) [2,3]. Obesity is associated with the dyslipidemia profile, including high blood triglyceride and low-density lipoprotein (LDL) levels [4,5], a direct risk factor for cardiovascular disease. According to previous studies, a fat-rich and carbohydrate-rich diet is an important factor leading to an excessive accumulation of lipids on white adipose tissue[6–8]. During energy metabolism pancreatic enzymes (lipase and alpha glycosidase) play a primary role in the digestion of fat (>90% mixed triglyceride) and digestible starch into a final product of fatty acid and glucose, respectively, followed by absorption [9]. The excess fatty acids are then re-synthesized into triglyceride and stored in adipose tissue while high blood glucose levels promote *de novo* lipogenesis in adipose tissue by a mechanism involving the transcriptional control of the SREBP1c and ChREBP transcription factors. As a result, excess glucose and fatty acid lead to an increase in both the size and number of adipocytes. Inhibition of digestive enzymes is therefore one of the most important routes for managing obesity. Decreasing energy-absorbing substances such as glucose and fatty acids from food has been seen to yield promising results for weight loss and decreased hypertriglyceridemia.

Studies in pharmaceutical interventions for weight management have produced results. Orlistat (anti-obesity drugs) appears to suppress digestive lipases as well as lipolysis levels, resulting in reduced fat accumulation. To treat obesity and hyperglycemia in people with type 2 diabetes, acarbose, miglitol, and voglibose have been used to block starch digestive enzyme activity in the gastrointestinal tract and limit the pace of starch digestion in order to prevent hyperglycemia [10]. These drugs often cause side effects like diarrhea and general discomfort because they work by blocking -amylase and -glucosidases, causing the undigested starch to be excreted in the colon [10]. An alternate method for regulating glucose absorption in the body while assuring more thorough starch digestion is to use weaker inhibitors of the starch-degrading enzymes [11]. Dysregulated fatty acid metabolism (an imbalance between increased fatty acid synthesis and impaired fatty acid oxidation) is reported to be a significant contributing factor leading to an increase of triglyceride, and low-density lipoprotein cholesterol (LDL-C), while decreasing levels of high-density lipoprotein cholesterol (HDL). Recently, *de novo* lipogenesis inhibitors reached the clinical approval process for the treatment of hyperlipidemia [12]. In a previous study by Gao 2019, rats given WZ66 (Acetyl-CoA carboxylase 1 (ACC1) and Acetyl-CoA carboxylase 2 (ACC2) inhibitor) showed a decrease in fatty acid synthesis compared to the control group [13]. It would appear, therefore, that the development of food digestion and absorption inhibitors is a promising approach for the pharmaceutical management of obesity. Future trend perspective on natural anti-obesity drugs and their therapeutic significance in the management of obesity.

According to WHO recommendations, plant-derived medicines consumed as part of a normal diet play an important role in promoting healthy weight management [14]. Plant-based compounds have been shown to possess anti-obesity properties and appear to work through regulation of the lipid profile by acting on multiple targets (i.e. digestive enzymes) and by altering *de novo* fatty acid synthesis [15]. Kratom for instance (of the Rubiaceae family), is used in traditional Thai and Malaysian self-treatment of pain and diabetes [16]. Moreover, the diversity of Kratom strains, their phytochemical composition, and possible nutritive values can vary based on numerous factors, such as soil conditions and climate. Nutritively, Kratom has shown potential benefits, particularly in the realm of animal nutrition. *Mitragyna speciosa* leaves have been noted to contain trace minerals and vitamins, including calcium, potassium, and magnesium. The presence of dietary fiber in Kratom can be inferred from its leafy nature [17]. According to reports among the those who regularly use Thai Kratom (*Mitragyna speciosa*), individuals have lower body mass index (BMI), serum triglyceride level, and serum LDL-C level when compared to non-users [18], suggesting that Kratom may indeed contain anti-obesity properties. Mitragynine (an alkaloid compound) was identified as the major phytochemical and showed biological activity such as targeting the mu-opioid receptor. However, more research is needed to document the molecular mechanism responsible. In this study, we aim to determine the inhibitory activities of Thai Kratom and the major compound on pancreatic digestive enzymes (i.e., alpha-glucosidase and lipase) and the ACC1 enzyme. Furthermore, modes of inhibition and *K*_i_ were carried out.

## Material and methods

### Materials

Mitragynine (Lipomed, Inc., lot number 1610.1B0.2) was kindly obtained from Assoc. Prof. Dr. Gorawit Yusakul, School of Pharmacy, Walailak University. Quercetin (Sigma-Aldrich, St Louis, MO, USA, Lot number Q4951), and rutin (Acros Organics, Lot number A0355330) α-Glucosidase (Sigma-Aldrich, St Louis, MO, USA) p-nitrophenol-α-D-glucopyranoside (*p*NPG) (Sigma-Aldrich, St Louis, MO, USA, Lot number 3698310) acarbose (positive control) (Sigma-Aldrich, St Louis, MO, USA, Lot number SLCF5122) orlistat (Sigma-Aldrich, St Louis, MO, USA, Lot number 0000117290).

### Plant material

Fresh leaves of *M*. *speciosa* (Kratom) were collected from Developing a Kratom Plant Control Model by using community participation in Thailand: a case study of Nam Pu Sub-district, Ban Na San district, Surat Thani province, Thailand (GPS: 8.7384933, 99.2657274) (Latitude: 8° 38’ 42.2” N; Longitude: 99° 53’ 47.6” E). Leaves of RTK and GTK were identified by Dr. Prateep Panyadee, Plant Varieties Protection, Department of Agriculture, Thailand. Voucher specimens of RTK (P. Panyadee 316) and GTK (P. Panyadee 317) were deposited at Queen Sirikit Botanic Garden (QBG), Thailand, in February 2022.

### Liquid chromatography analysis of kratom extracts

**Fig 1:**
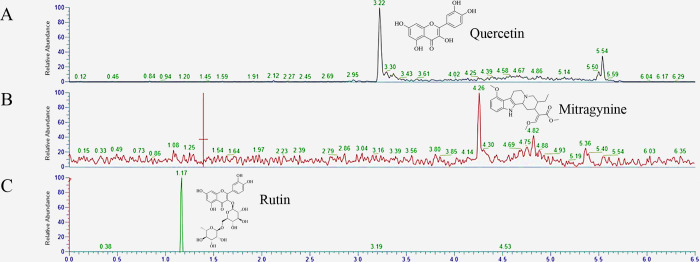
Chromatograms of quercetin (A), mitragynine (B), and rutin (C) were obtained from the Kratom crude ethanol extract. A 10 μg sample of the Kratom crude ethanol extract was separated using C18 column chromatography, with the elution solvents being a mixture of water and acetonitrile. Quercetin (A), mitragynine (B), and rutin (C) were eluted at 3.22, 4.26, and 1.17 minutes, respectively.

The major compounds in Kratom were determined using UHPLC model Ultimate 3000 with LC-MS/MS model Altis Plus (Thermo Fisher Scientific, MA, USA) as the previous descriptive in [19]. After samples were filtered through a nylon filter with a pore size of 0.22 μm, 10 μl of sample was subjected to separation using Thermo Hypersil GOLD C-18 (2.1 × 100 mm, 1.9 μm) as the stationary phase, and the mobile phase was a mixture of solvent A: 0.1% formic acid (pH 2.99), and solvent B: acetonitrile. The elution condition was set as 0–0.5 min, 25% B; 3–4 min, 80–100% B; and 4–6.5 min, 25% B, with a flow rate of 0.5 mL/min. The liquid chromatography was in tandem with a mass analyzer which scanned mass to chart ratios ranging from 100 to 1,700 m/z in positive mode. Mitragynine (m/z 399.20 → 174.10), quercetin (m/z 303.05 → 229.00), and rutin (m/z 611.16 → 303.10) were eluted at 2.08, 1.20, and 1.22 min, respectively (Fig 1). Pure compounds of mitragynine, quercetin, and rutin were obtained from Lipomed, Inc., (lot number 1610.1B0.2), Sigma-Aldrich, St Louis, MO, USA, (Lot number Q4951), and Acros Organics (Lot number A0355330), respectively, for further experiments.

### Inhibition effect of tests inhibitor on α-glucosidase activity

Inhibition of alpha-glucosidase was performed using *p*-nitrophenol-α-D-glucopyranoside (*p*NPG) as a substrate. Alpha-glucosidase hydrolyzed *p*NPG with *K*_m_ and *V*_max_ values of 0.18 mM and 28.24 μmol/min/mg protein is in range with the previous report [20]. In the 150 μl final reaction volume, 0.1 unit of α-Glucosidase was incubated with test inhibitors (crude ethanol extract (final concentration 0–100 μg/mL), pure compounds (mitragynine, quercetin, and rutin (final concentration 0–100 μM at 37°C in 0.1 M sodium phosphate buffer (pH = 7.4)). After 10 min, the reactions were started by adding 50 μL *p*NPG at *K*_m_ (0.18 mM). The rate of alpha-glucosidase-mediated *p*NPG was determined by measuring the *p*-nitrophenol product at 405 nm (a microplate reader (Multiskan SkyHigh, Thermo Scientific, Göteborg, Sweden)) against time (10 min). The IC_50_ was determined by plotting the remaining activity of alpha-glucosidase at each inhibitor concentration and test inhibitor concentration. IC_50_ was calculated by fitting curves to non-linear regression (GraphPad Prism version 9.3.1), and acarbose was used as the positive control. *K*_i_ and mode of inhibition were further determined for pure compounds (mitragynine and quercetin). Briefly, 0.1 U α-Glucosidase enzyme were incubated with test inhibitors (mitragynine (0, 64, 128, 256 μM); quercetin (0, 2.5, 5, 10 μM); acarbose (0, 0.5, 1, 2 mM)) at 37°C. After 10 min, the reactions were started with the addition of *p*NPG at 0.007, 0.015, 0.03, 0.06, 0.125, 0.25, and 5 mM. The mode of inhibition and inhibition constant (*K*_i_) were determined by Lineweaver-Burk plots.

### Inhibition effect of tests inhibitor on lipase activity

Inhibition of lipase was performed using *p*-nitrophenyl palmitate (*p*NPP) as a substrate. Lipase hydrolyzed *p*NPP with *K*_m_ and *V*_max_ values of 1.3 mM and 2.3 μmol/min/mg protein is in range with the previous report [21]. In the 150 μl final reaction volume, 0.1 unit of lipase was incubated with test inhibitors (crude ethanol extract (final concentration 0–100 μg/mL), hexane fraction, EtOAc, and aqueous fraction (final concentration 0–100 μg/mL), and pure compounds (mitragynine, quercetin, and rutin (final concentration 0–100 μM at 37°C in 15 mM Tris-HCl buffer (pH 8.0). After 10 min, the reactions were started by adding 50 μL *p*NPP at *K*_m_ (1.3 mM). The rate of lipase-mediated *p*NPP was determined by measuring the p-nitrophenol product at 405 nm (a microplate reader (Multiskan SkyHigh, Thermo Scientific, Göteborg, Sweden)) against time (10 min). The IC_50_ was determined by plotting the remaining activity of lipase at each inhibitor concentration and test inhibitor concentration. IC_50_ was calculated by fitting curves to non-linear regression (GraphPad Prism version 9.3.1.), and orlistat was used as the positive control. *K*_i_ and mode of inhibition were further determined for pure compounds (mitragynine and quercetin). Briefly, 0.1 U lipase enzyme were incubated with test inhibitors mitragynine (0, 15, 30, 60 μM), quercetin (37, 75, 150 μM), orlistat (0, 2.5, 5 μM) at 37°C. After 10 min, the reactions were started by the addition of *p*NPP at 0.02, 0.03, 0.06, 0.125, 0.25, 3.5 and 5 mM. The mode of inhibition and inhibition constant (*K*_i_) were determined by Lineweaver-Burk plots.

### Inhibition effect of tests inhibitor on ACC1 activity

An ACC1 inhibition assay was performed using the ACC1 Assay Kit from BPS Bioscience (USA) following the manufacturer’s protocol. Mitragynine and quercetin were tested at concentrations of 0, 1, 10, and 100 μM, and incubated with 3.75 μl of Acetyl-CoA, 11.25 μl of Sodium Bicarbonate, and 15 μl of ATP in a total reaction volume of 25 μl. After a 10-minute pre-incubation period, the reaction was initiated by adding 0.1 ng/mL of ACC1 enzyme. The reactions were allowed to proceed for 40 minutes before being terminated by the addition of 100 μl of chilled acetonitrile. The samples were then centrifuged, and the supernatants were carefully transferred to new tubes for analysis. The rate of reaction was quantified by monitoring the decrease in acetyl-CoA substrate concentration using a C18 HPLC column, employing a mobile phase consisting of a linear gradient of water and methanol.

### Determination of lipid accumulation by Oil Red O staining

The differentiation of 3T3-L1 adipocyte cells was performed according to Ngamdokmai et al., 2022. Briefly, 3T3-L1 adipocyte cells were cultured in DMEM supplemented with 10% (v/v) FBS, 0.5 mM IBMX, 1 μM DEX, and 10 μg/mL insulin (INS) for 48 hours. The effect of GTK and RTK on lipid accumulation was performed by incubation of GTK and RTG crude ethanol extract (0–10 μg/mL) with 3T3-L1 adipocyte cells. After every 24 hours (for 3 days), the media was removed and replaced with DMEM supplemented with 10% (v/v) FBS, 0.5 mM IBMX, 1 μM DEX, and 10 μg/mL insulin (INS) containing 0, 4, 6, 8 and 10 μg/ml GTK and RTK crude ethanol extract. At the end of day 3 of differentiation, the 3T3-L1 cells were washed twice with PBS and incubated with paraformaldehyde in a final volume of 1 mL for 30 min. After paraformaldehyde was removed and cells were rinsed twice with PBS, the 3T3-L1 cells were stained with filtered Oil Red O solution for 1 h. 3T3-L1 cells were then washed twice with PBS and were photographed under an inverted microscope (Axiovert 40 CFL, Carl Zeiss, Jena, Germany). The resulting red-stained lipid droplets were extracted with 100% isopropanol. The absorbance was measured at a wavelength of 520 nm to quantify lipid accumulation within the adipocytes.

### Statistical analysis

All data were obtained from three dependent experiments and are expressed as the mean ± standard deviation (SD). Statistical analysis was performed using GraphPad Prism 9.3.1 (GraphPad Software Inc., San Diego, California, USA) with one-way ANOVA. Differences with *p* < 0.05 were statically significant.

## Results

### Inhibition-gilded assay for Isolation of active compound in Thai Kratom leaves

Regular use of Thai Kratom has been reported to lower blood triglyceride and BMI among healthy people when compared to non-use [17]. We hypothesized that Thai Kratom and its phytochemical compounds might alter the activity of pancreatic digestive enzymes (lipase and alpha-glucosidase). To test this hypothesis, GTK and RTK were collected from the Developing a Kratom Plant Control Model by using community participation in Thailand: a case study of Nam Pu Sub-district, Ban Na San district, Surat Thani province, Thailand, which reported their traditional use. The ground and dried powder of RTK and GTK were extracted using ethanol. LC-MS/MS analysis was performed to determine the major compounds present in the ethanol extracts of both RTK and GTK. The result indicated that GTK contains mitragynine levels (the major alkaloid compound) 2 times higher than RTK (Fig 1, Table 1), whereas quercetin and rutin are found in nearly the same amount, ranging from 10.2–17.4 mg/g (EtOH extract) (Fig 1, Table 1).

**Table 1:** The concentration levels of mitragynine, quercetin, and rutin in Kratom ethanol extract.

Sample	Concentration of compounds: mean±SD (mg/g)		
	Rutin	Quercetin	Mitragynine​
Green Thai Kratom ETOH extract	10.20±2.95 (1.00%w/w)	17.40±5.02 (1.70%w/w)	63.60±18.34 (6.30%w/w)
Red Thai Kratom ETOH extract	16.60±4.78 (1.60%w/w)	17.10±4.91 (1.70%w/w)	36.70±10.59 (3.60%w/w)

### Inhibition effect of mitragynine, quercetin, and rutin on alpha-glucosidase and lipase activity

**Fig 2:**
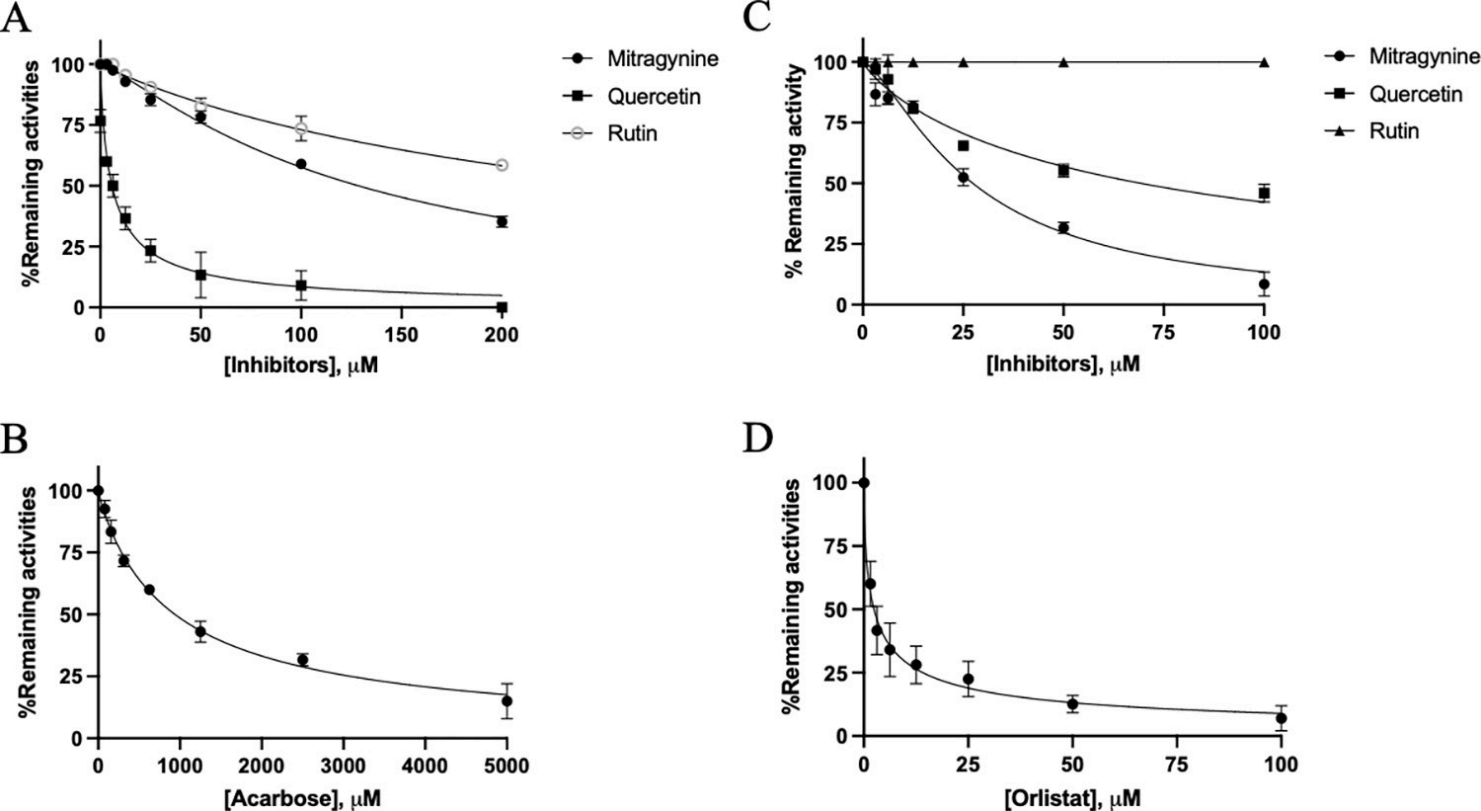
Inhibitory effect (IC_50_) of quercetin, mitragynine, and rutin on alpha-glucosidase (A) and lipase (B) activity. Acarbose (C) and orlistat (D) were used as the positive inhibitor.

We next determined the inhibitory effect of RTK and GTK crude ethanol against alpha-glucosidase and lipase activity. The results showed that the GTK EtOH extract produced stronger effective inhibition of lipase activity than alpha-glucosidase activity with IC_50_ values of 22.58 ± 1.75 μM and 42.00 ± 4.24 μM, respectively (Table 2). Mitragynine (major compound) showed a stronger inhibitory effect on lipase than alpha-glucosidase, with IC_50_ values of 27.40 μM (11.3x higher than orlistat (IC_50_ = 2.41 μM) and 128.60 μM (7.3-fold lower than acarbose (IC_50_ = 938.40 μM), respectively (Fig 2). Quercetin exhibited strong inhibition of alpha-glycosidase, with an IC_50_ value of 5.80 μM, however it only produced weak inhibition of lipase (IC_50_ = 69.28 μM). Relative to mitragynine and quercetin, rutin showed weak inhibition of both enzymes, with IC_50_ more than 100 μM (Fig 2, Table 2).

**Table 2:** Inhibitory effect of Kratom extract and major compounds on alpha-glucosidase and lipase.

Test inhibitors	Half-maximal inhibitory concentration (IC_50_)	
	Alpha-glucosidase	Lipase
Crude EtOH extract		
Thai Green Kratom	42.00±4.24	22.58±1.75
Thai Red Kratom	51.29±5.44	40.88±0.024
Major compound		
Mitragynine	128.60±5.03	27.40±2.90
Quercetin	5.82±0.95	69.28±3.04
Rutin	283.40±10.23	>100
Positive control		
Orlistat	NA	2.41±0.58
Acarbose	938.40±35.28	NA

**Fig 3:**
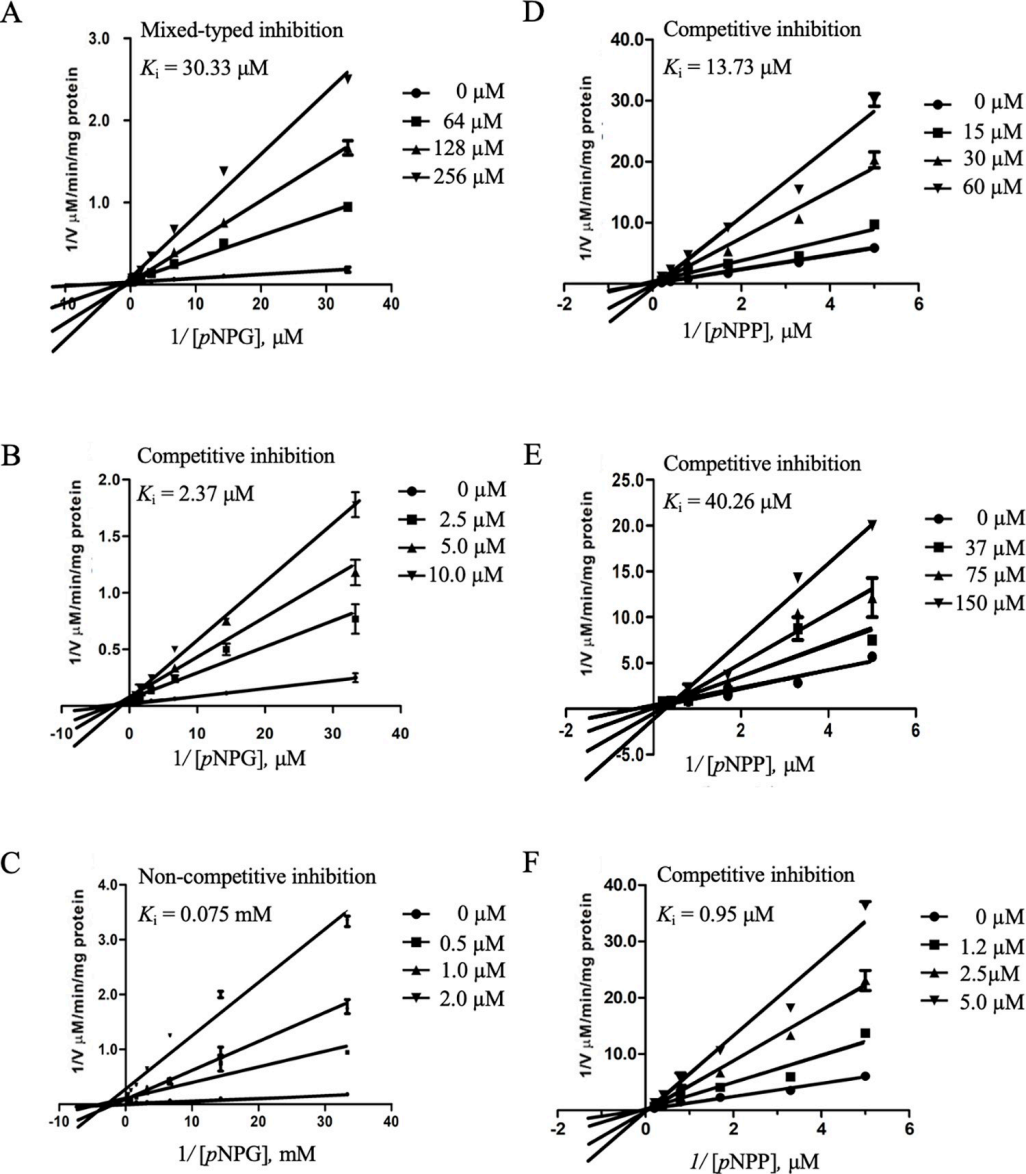
The Lineweaver-Burk plots for the mode of inhibition and Ki values of alpha-glucosidase-mediated *p*NPG and lipase-mediated *p*NPP by mitragynine (A), quercetin (B), and acarbose (C), as well as mitragynine (D), quercetin (E), and orlistat (F), respectively. The experiments were done in triplicate.

This result indicates that mitragynine and quercetin were selective inhibitors for lipase and alpha-glucosidase, respectively, and could block triglyceride and starch digestion and absorption. Consequently, mitragynine and quercetin were selected for a follow-up experiment to determine the mode of inhibition and kinetic constant (*K*_i_). According to Lineweaver Burk plots, mitragynine induced competitive and mix-typed inhibition against lipase (*K*_i_ = 13.73 μM) and alpha-glucosidase (*K*_i_ = 30.33 μM), respectively. However, quercetin was competitive for inhibition of both lipase and alpha-glycosidase, with *K*_i_ values of 40.26 and 2.37 μM, respectively. Mitragynine was a weak inhibitor when compared to orlistat (positive inhibitor, *K*_i_ 0.95 μM), whereas mitragynine was a strong inhibitor for alpha-glucosidase (*K*_i_ = 75 μM) (Fig 3A–3F).

### Combination inhibition of mitragynine, quercetin, and acerbose on alpha-glycosidase activity

**Fig 4:**
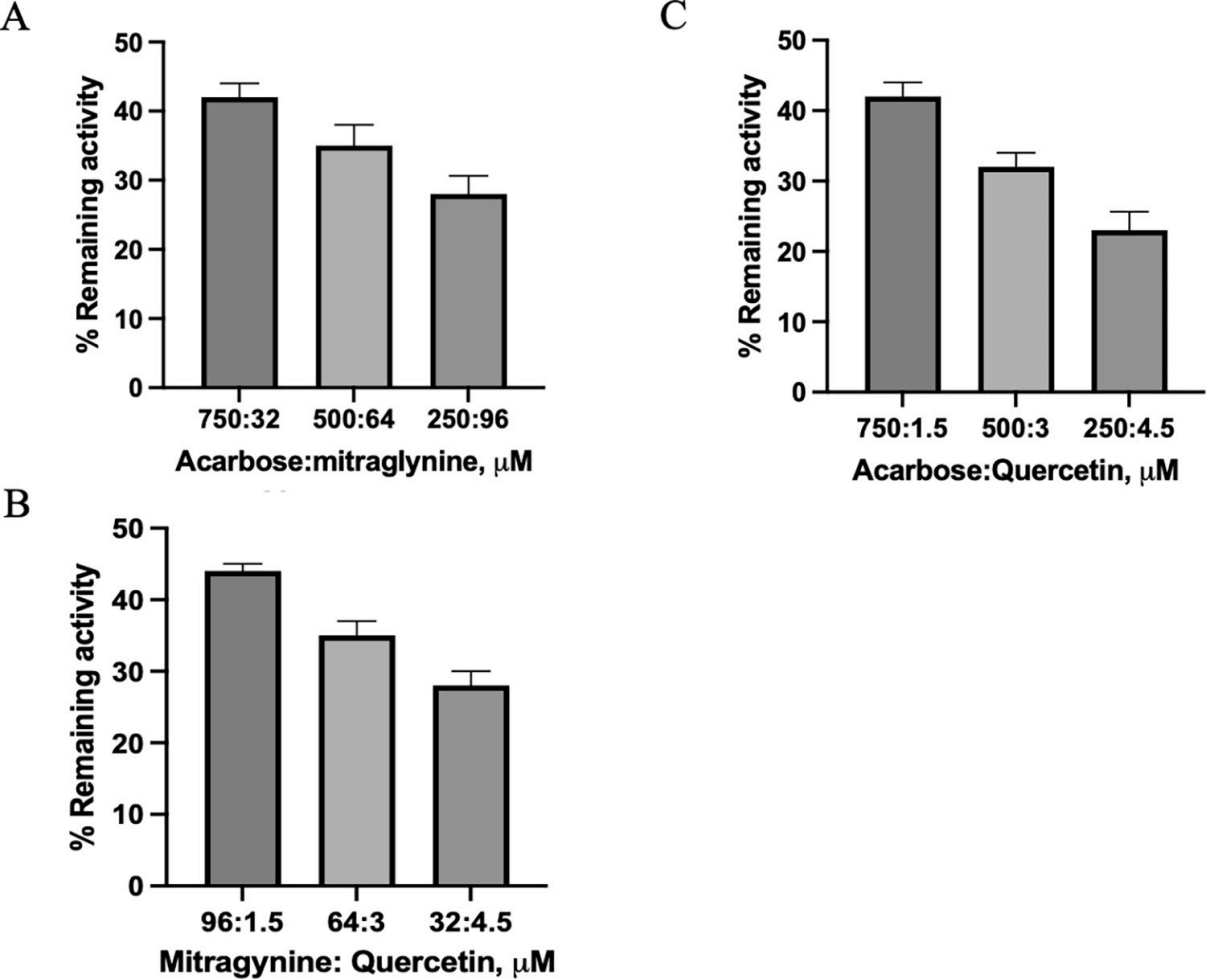
The simultaneous inhibition of acarbose:mitragynine (A), acarbose:quercetin (B), and mitragynine:quercetin (C) on alpha-glucosidase-mediated *p*NPG. The experiments were done in triplicate.

According to the different modes of inhibition of Mitragynine, quercetin, and acarbose on alpha-glycodidase enzyme activity, we hypothesized that these compounds might work as inhibitors with synergist effect. To test this hypothesis, combination inhibition between acarbose and mitragynine, acarbose and quercetin, and mitragynine and quercetin, was performed. The result demonstrated that these compounds had a synergistic effect on alpha-glycosidase-mediated *p*NPG. The increasing ratio of quercetin increased the inhibition of the alpha-glycosidase enzyme. Acarbose and quercetin, in the ratio of 250 and 4.5 μM, showed stronger inhibition when compared to acarbose and quercetin at IC_50_ alone. Moreover, the synergistic effect of mitragynine and quercetin indicated that intakes of GTK and RTK containing mitragynine and quercetin might decrease the rate of starch digestion and absorption (Fig 4A–4C).

### Effect of GTK and RTK on fat accumulation through inhibition of ACC1 activity

**Fig 5:**
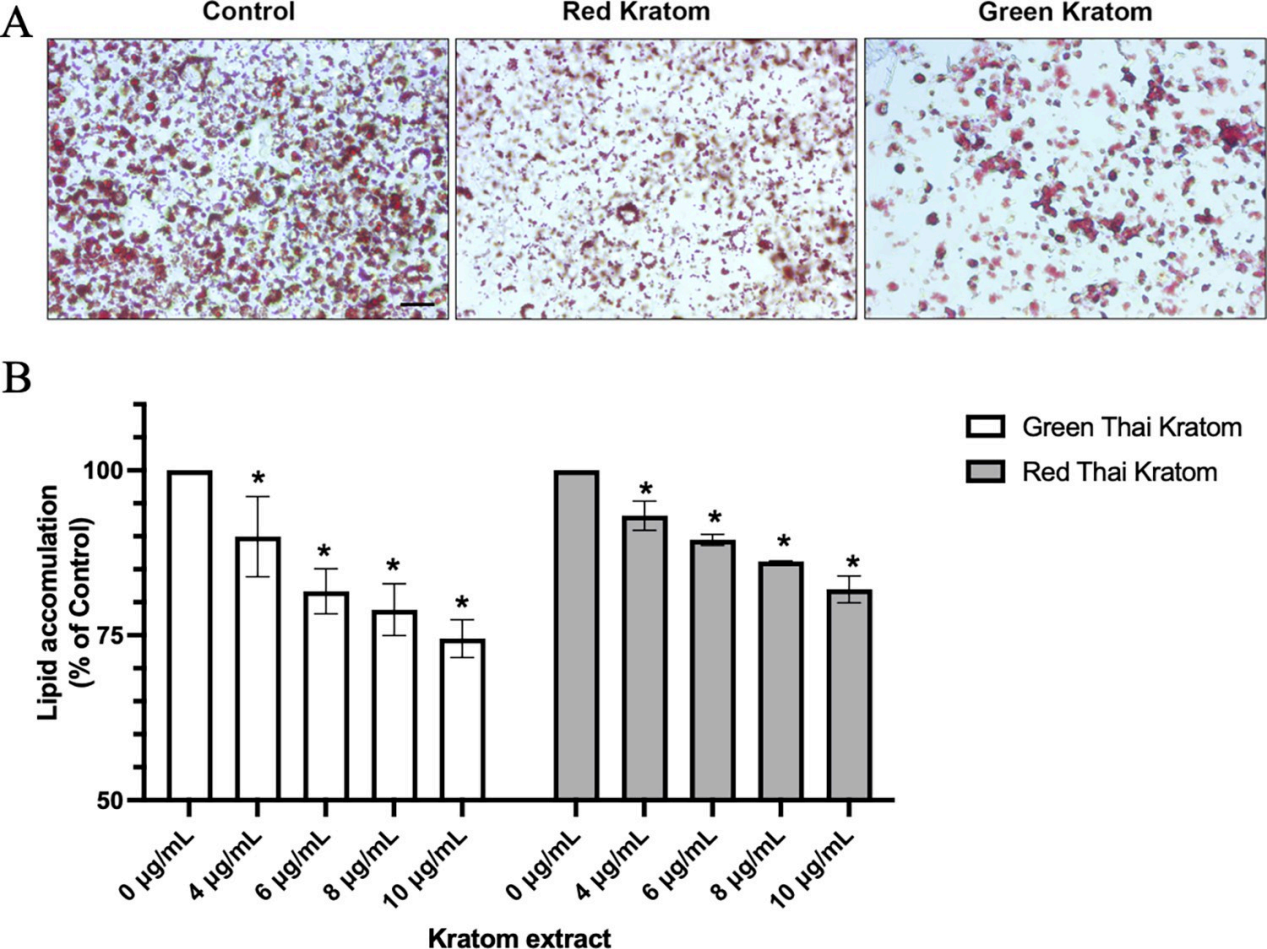
Effect of Red and Green Kratom on fat accumulation in 3T3-L1 cells. After 72 hours, lipid accumulation in 3T3-L1 cells treated with Red and Green Kratom (0–10 μg/mL) was determined using Oil Red O staining (A). Lipid accumulation at each concentration was assessed in triplicate experiments and determined by measuring the absorbance of Oil Red O at 500 nm (B). The asterisk (*) indicates a statistically significant decrease in fat accumulation compared to 0 μM of each test inhibitor (p < 0.05, Student’s t-test). The experiments were done in triplicate.

**Fig 6:**
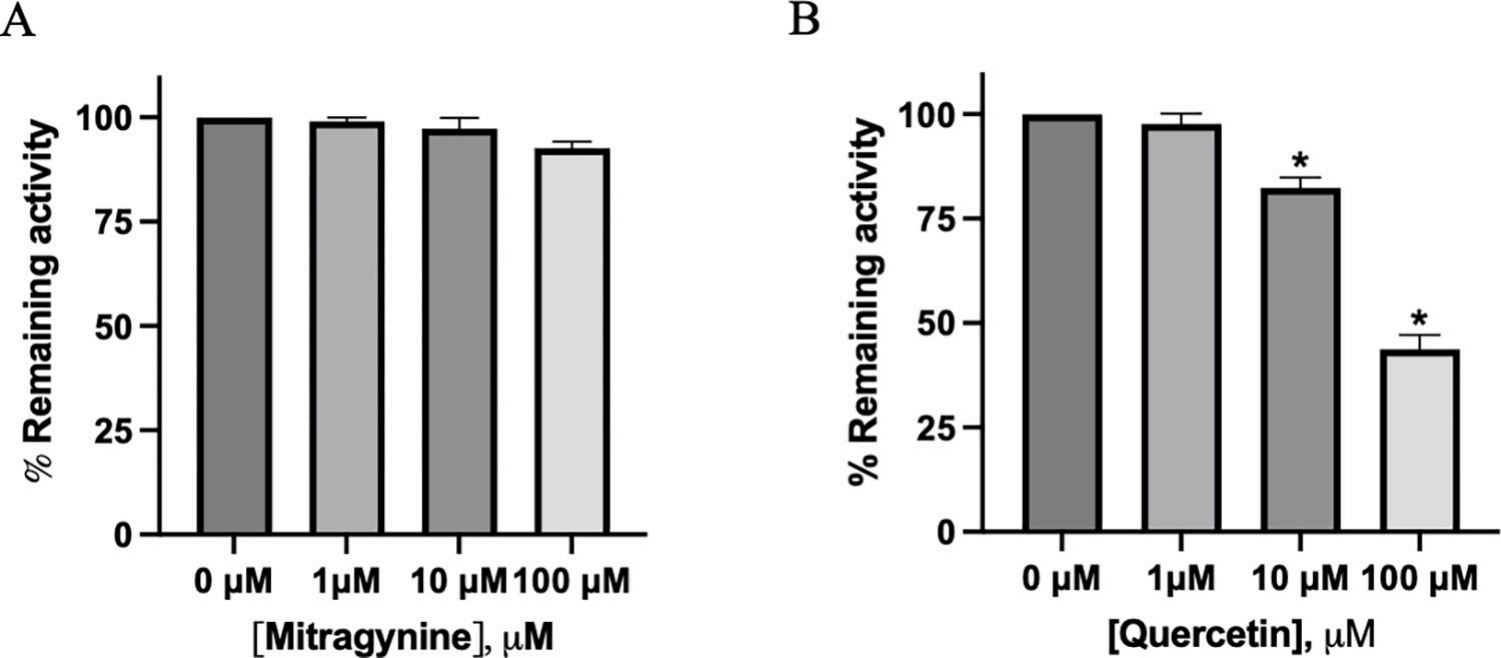
Effect of mitragynine (A) and quercetin (B) against ACC1 activity. The remaining activity of ACC1 was determined by the decrease in acetyl-CoA substrate, and the experiments were performed in triplicate. The asterisk (*) indicates a statistically significant decrease in remaining ACC1 activity compared to 0 μM of each test inhibitor (p < 0.05, Student’s t-test). The experiments were done in triplicate.

To determine whether GTK and RTK contain anti-obesity properties, 3T3-L1 adipocyte cells were treated with GTK and RTK in different concentrations ranging from 0–10 μg/mL, which is lower than the toxic concentration. After 72 hours of treatment, fat accumulation was determined using Oil Red O staining, and the result demonstrated that both GTK and RTK decrease fat accumulation in a dose-dependent manner (Fig 5A). At 10 μg/mL GTK and RTK decrease fat accumulation by 25% and 17% respectively when compared to vehicle control (Fig 5B), indicating that GTK and RTK crude ethanol extract might contribute to triglyceride synthesis in 3T3-L1 adipocyte cells. We then determined the effect of mitragynine (the major compound) and quercetin on ACC1 activity, which is the rate-limiting step in *de novo* fatty acid biosynthesis. To test this, inhibition of ACC1 activity was performed with an ACC1 activity kit. We found that quercetin, but not mitragynine, decreased ACC1 activity in a dose-dependent manner. At 100 μM of quercetin, the activity of ACC1 decreased by 60%, indicating that inhibition of ACC1 by quercetin might decrease fat accumulation in 3T3-L1 adipocyte cells (Fig 6, S1 Fig).

## Discussion

Obesity is considered a major risk factor for type 2 diabetes and cardiovascular disease, affecting both mortality and quality of life. Dietary patterns containing high levels of fat and carbohydrates has been linked to obesity, which increases the prevalence and size of adipose tissue. In energy metabolism, digestion and the absorption of fatty acid and glucose are the primary steps in the oxidation of nutrients to energy production. The high-fat absorption leads to fat accumulation in adipose tissue. Moreover, high glucose concentration will increase de novo fatty acid biosynthesis. Taken together, high intake of fatty acid and glucose leads to an increase of adipocyte cell in both number and size. The dysregulation of adipocyte cells causes an increase in both triglyceride and LDL-C, which is the risk factor for cardiovascular disease and obesity-induced insulin resistance. According to WHO recommendations, plant-food therapy is the first line in the prevention of obesity. Inhibition of pancreatic enzymes (both lipase, and alpha-glucosidase) has been reported to be one of the drug targets for anti-obesity.

In a previous report by La-Up *et*. *al*., 2022 [18], Thai Kratom was shown to decrease BMI, blood triglyceride, and LDL among regular users in Developing a Kratom Plant Control Model by using community participation in Thailand: a case study of Nam Pu Sub-district. However, the molecular mechanism responsible for improving lipid profile and the anti-obesity mechanism is less well documented. In this study, GTK and RTK were collected based on self-reported use in Num Phu Charter. We first determined the major constituents in both GTK and RTK. The results indicated that mitragynine (the major compound) was 63.6 and 36.8 mg/g of crude ethanol extract respectively, and these values are comparable to the report of Prozialeck, 2020 found mitragynine concentrations between 3.9–62.1 mg/g in Kratom samples [22]. Quercetin and rutin were approximately 1 mg/g.

Mitragynine showed effective inhibition of lipase-mediated *p*NPP with IC_50_ and *K*_i_ values of 22 μM and 10 μM, respectively, in the competitive mode of inhibition. The IC_50_ of mitragynine was comparable to mahanimbine compound isolated from *Murraya koenigii* leaves. Mahanimbine inhibited lipase activity with an IC_50_ value of 17.9 μM (S2 Fig). Both mitragynine and mahanimbine are indole alkaloid compounds containing a structural moiety of indole [23]. In contrast to mitragynine, quercetin (69.28 μM) and rutin (> 100 μM) showed weak inhibition toward lipase activity. This finding was similar to a previous report by Zhou et al. 2022 which investigated the inhibitory effect of quercetin on lipase activity and plasma triglyceride concentration in HFD-induced rats. Quercetin inhibited lipase activity with IC_50_ 70 μg/ml (~230 μM), resulting in blockage of triglyceride ingestion and absorption *in vivo*. In the quercetin-treated group, plasma triglyceride was observed to be lower than in the control group [24]. It might therefore be possible to explain why people who regularly use GTK and RTK have lower plasma triglyceride than non-users. Although glucose plays a primary role in energy production for humans, excess glucose leads to the conversion of glucose into fatty acid through *de novo* lipogenesis, resulting in excessive expansion of white adipose tissue and obesity. The control of glucose uptake might prevent the generation of fatty acid from glucose (*de novo* lipogenesis).

We next determined the effect of major compounds found in Thai Kratom towards the alpha-glucosidase activity of which this enzyme hydrolyzes disaccharide into glucose, before glucose absorption. We found quercetin to be a strong and effective inhibitor of alpha-glucosidase activity, with IC_50_ value of 5.6 μM. This is consistent with a previous report by Lim et al., 2019 which indicated that the quercetin-containing hydroxyl group at 3C of C ring showed the strongest inhibition against alpha-glucosidase enzymes when compared to lutein and eriodictyol [25]. Rutin at C3 of C ring is substituted by rhamnoglucoside in state of the hydroxy group. We believe this might be the reason why rutin was a weaker inhibitor than quercetin in this study. Mitragynine in this study showed weak inhibition, with an IC_50_ value of 128.6 μM. In the previous study *O*-methyl mahanine and mahanine (indole alkaloid) both displayed inhibitory activity against alpha-glucosidase, with IC_50_ values of 46.1 μM and 21.4 μM respectively, indicating that the hydroxy group but not *O*-methyl group on moiety of indole led to a decrease in IC_50_ (S3 Fig). The hydroxy group, therefore, is crucial for an H-bonding donor to interact with alpha-glucosidase, while the methoxy group might potentially interrupt H-bonding formation [26]. However, mitragynine containing an *O*-methyl group on moiety of indole might not interact well with alpha-glucosidase.

According to the Lineweaver–Burk plot (Fig 3), *K*_i_ of mitragynine, quercetin, and acarbose was 13.73, 40.26, and 0.95 respectively, in different modes of inhibition. We attempted to test whether mitragynine, quercetin, and acarbose exhibited synergistic inhibition. To that end, combination inhibition of acarbose+mitragynine, acarbose+quercetin, and mitragynine+quercetin toward alpha-glucosidase was explored. Our results indicated that quercetin and acarbose yielded the strongest synergistic inhibition, at a ratio 0.25: 0.75 (acarbose (250 μM): quercetin (4.5 μM)). This result is similar to that previously reported by Koh, 2022 that the combination of acarbose and quercetin produced synergistic inhibition which was stronger inhibition than either alone [27]. Moreover, the different binding sites of mitragynine (mix-typed inhibition) and quercetin (competitive inhibition)—the major compounds in GTK and RTK—might promote co-inhibition, wrestling in synergistic inhibition. As a result, inactivation of alpha-glucosidase by GTK and RTK could decrease the rate of alpha-glucosidase-hydrolyzed glucose and prevent an excess amount of glucose from entering blood circulation.

In an experimental animal model subjected to a high-fat high-sucrose diet, plant extract was found to increase lifespan and prevent adipose tissue dysfunction. Adipose tissue dysfunction is one of the causes of metabolic disorders including insulin resistance. It is for this reason that adipose tissue was the focused therapeutic target for reducing body weight. In this study, 3T3-L1 adipocytes (*in vitro* adipocyte cell model) were incubated with GTK and RTK crude ethanol extract (0–10 μg/ml). After 72 hours, fat accumulation was determined using Oil Red O staining. Herein we thus reported for the first time that GTK and RTK decreased fat accumulation in 3T3-L1 adipocytes. Our study demonstrated that quercetin effectively inhibited ACC1, which plays a crucial role as the rate-limiting enzyme in fatty acid biosynthesis (Fig 6). This finding is consistent with previous reports indicating that quercetin compounds effectively inhibit ACC1 activity at varying concentrations [28]. Recent studies have shown that quercetin, rather than mitragynine, displays potent inhibition of ACC1, which plays a crucial role in regulating fatty acid biosynthesis. In contrast to these findings, previous studies have demonstrated that alkaloid compounds have a strong inhibitory effect on ACC1, with IC_50_ values ranging from 0.001 to 5 μM. The structural features of MK-4074 are specifically designed to interact with the active site of the BC domain of ACC, thereby preventing the binding of substrates that are required for the carboxylation reaction to occur [29]. Additionally, the flexible linker region of MK-4074 enables it to adapt to the dynamic conformational changes that occur in the active site of the enzyme during the catalytic cycle [12,29]. It is possible that the lack of a flexible linker region in mitragynine may contribute to its weak inhibitory effect against ACC1. However, further investigation is required to determine the precise mechanisms underlying the differential inhibitory effects of quercetin and mitragynine on ACC1, including their effects on transcriptional controls of ACC1 mRNA and protein expression, such as SREBP1c and ChREBP. Our study also highlights the need for further investigation into the effects of mitragynine on SREBP1c and ChREBP, which are the transcriptional regulators of ACC1 mRNA and protein expression. By elucidating the mechanisms of action of mitragynine on these transcriptional controls, we can better understand the potential benefits of mitragynine as a therapeutic target in metabolic disorders associated with dysregulated fatty acid synthesis.

## Conclusion

GTK and RTK contain mitragynine and quercetin as their major phytochemical compounds. GTK contains levels of mitragynine higher than RTK, with values of 63 and 36 mg/g respectively, whereas quercetin levels were nearly the same in both GTK and RTK. Mitragynine and quercetin selectively inhibit lipase (IC_50_ = 22 μM, *K*_i_ = 7 μM competitive mode) and alpha-glycosidase (IC_50_ = 128 μM, *K*_i_ = 75 μM mixed-type inhibition mode), respectively, resulting in a decrease of fatty acid and glucose absorption and protecting against fat accumulation. We report for the first time that Thai Kratom reduced *de novo* fatty acid synthesis through ACC1 inhibition, leading to a decrease in fat accumulation in 3T3-L1 adipocytes. Taken together, the regular use of Thai Kratom among certain Thai populations might lead to improved levels of blood triglyceride and lower BMI through inhibition of lipase, alpha-glucosidase, and ACC1 activity. Clinal trials are needed to determine effective dose, duration of action, lower toxic concentration, and side effects of Kratom use.

## Supporting information

S1 Fig Inhibitory effect of mitragynine and quercetin against ACC1-medated Accetyl-CoA. (TIF)

S2 Fig IC_50_ of indole alkaloid against lipase [23]. (TIF)

S3 Fig IC_50_ of indole alkaloid against alpha-glucosidase [26]. (TIF)
