# Correction: Effect of Green and Red Thai Kratom (*Mitragyna speciosa*) on pancreatic digestive enzymes (alpha-glucosidase and lipase) and acetyl-carboxylase 1 activity: A possible therapeutic target for obesity prevention

**DOI:** 10.1371/journal.pone.0305988

**Published:** 2024-06-18

**Authors:** Atikarn Janthongkaw, Sirinthip Klaophimai, Tanaporn Khamphaya, Supaporn Yimthiang, Yilin Yang, Ruixue Ma, Apirak Bumyut, Phisit Pouyfung

The third author’s name is spelled incorrectly. The correct name is: Tanaporn Khamphaya. The correct citation is: Janthongkaw A, Klaophimai S, Khamphaya T, Yimthiang S, Yang Y, Ma R, et al. (2023) Effect of Green and Red Thai Kratom (Mitragyna speciosa) on pancreatic digestive enzymes (alpha-glucosidase and lipase) and acetyl-carboxylase 1 activity: A possible therapeutic target for obesity prevention. PLOS ONE 18(9): e0291738. https://doi.org/10.1371/journal.pone.0291738.
