# Supplementary figures and images for: Effect of Green and Red Thai Kratom (Mitragyna speciosa) on pancreatic digestive enzymes (alpha-glucosidase and lipase) and acetyl-carboxylase 1 activity: A possible therapeutic target for obesity prevention
Source: PLoS One. 2023 Sep 21;18(9):e0291738. doi: 10.1371/journal.pone.0291738 (PMC10513218; doi:10.1371/journal.pone.0291738)

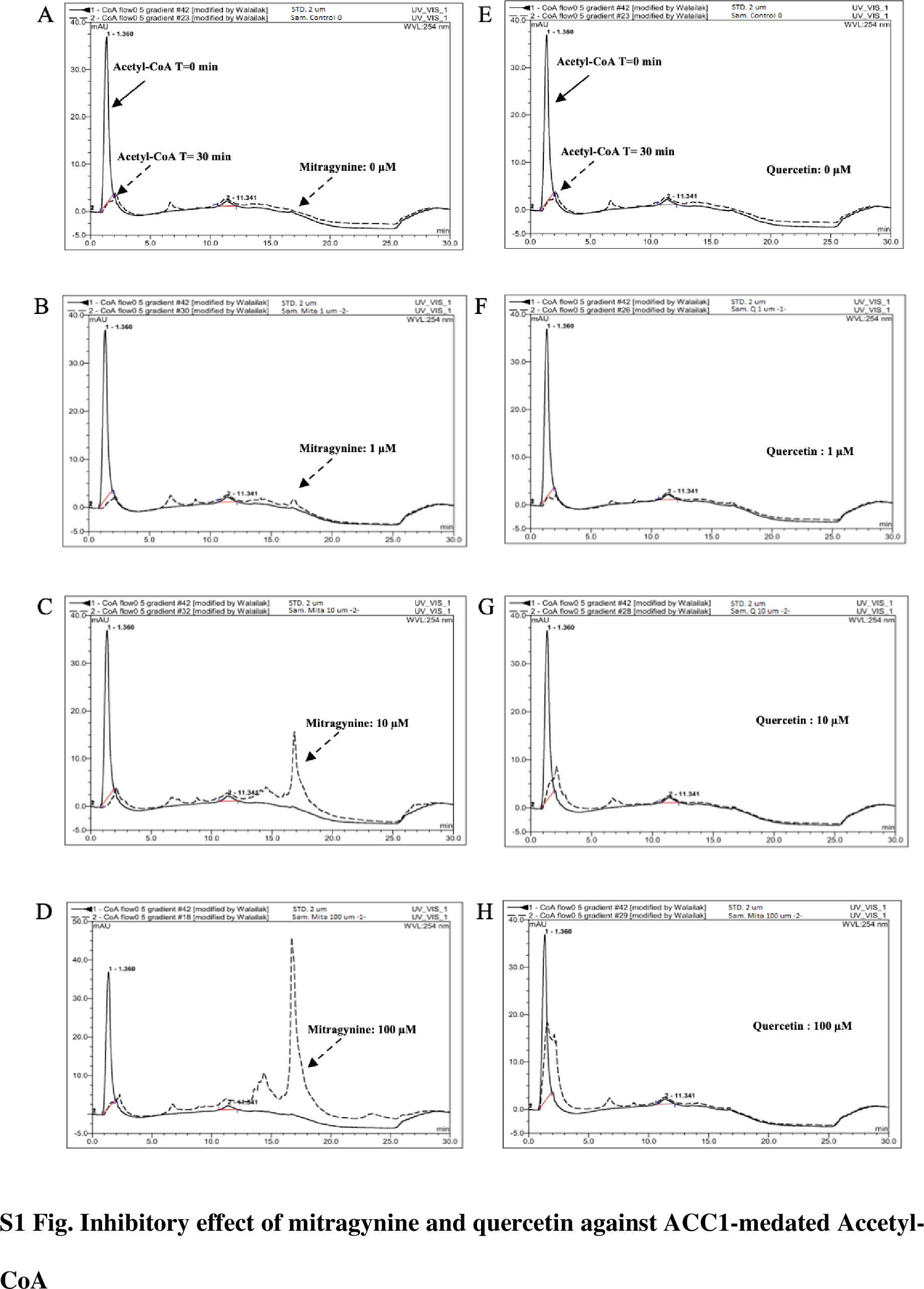

Supplement: S1 Fig — (TIF) [file pone.0291738.s001.tif]

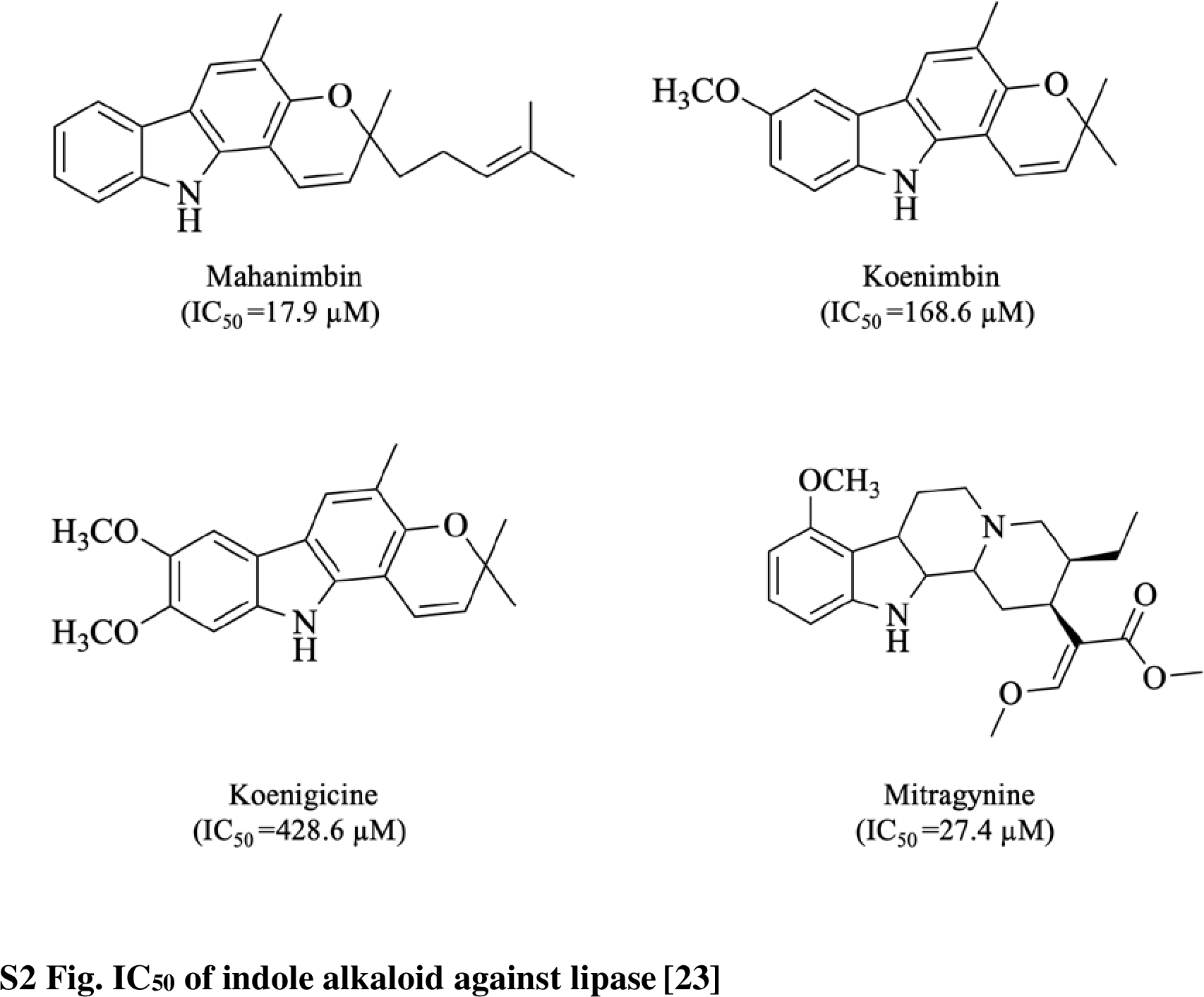

Supplement: S2 Fig — (TIF) [file pone.0291738.s002.tif]

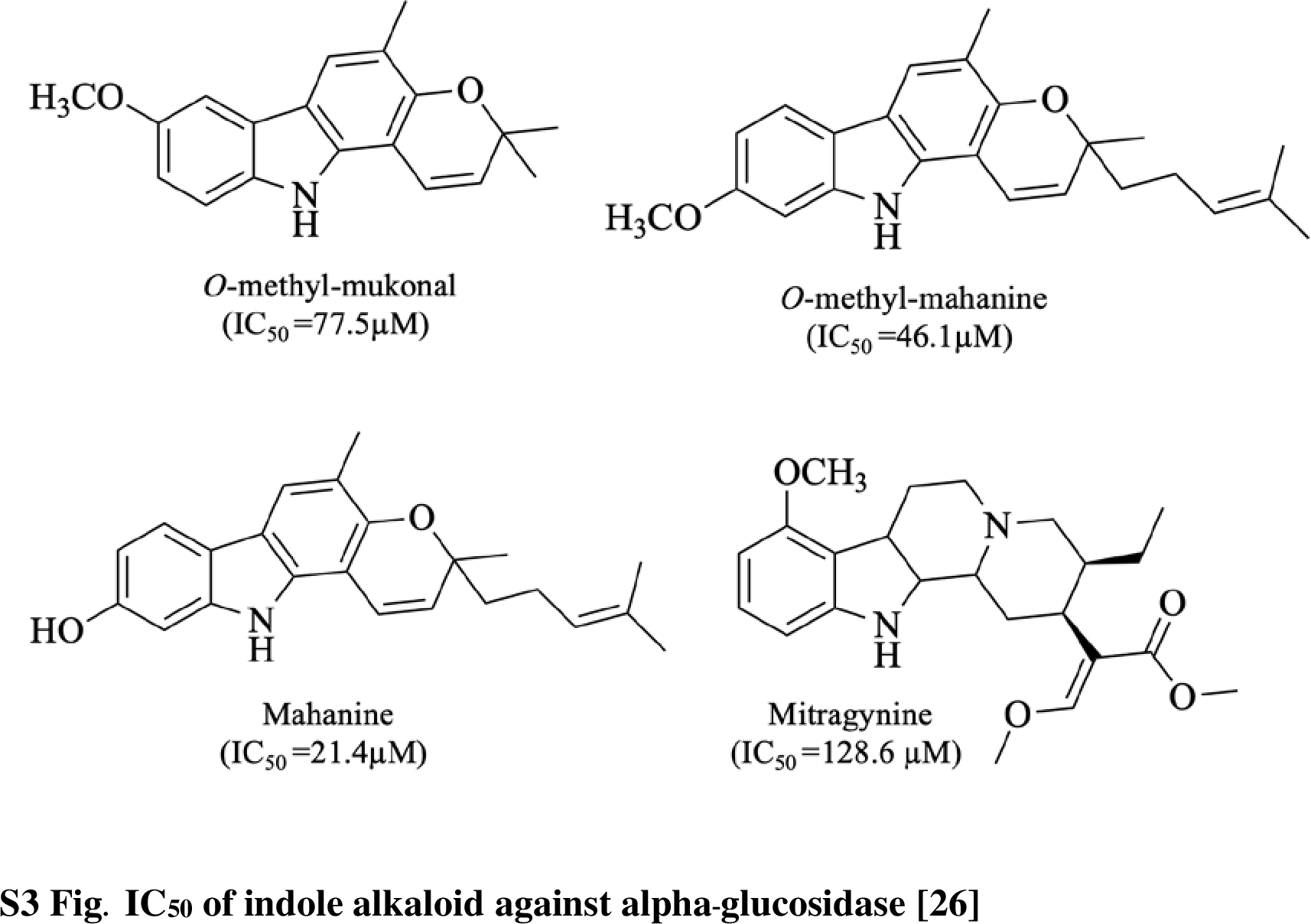

Supplement: S3 Fig — (TIF) [file pone.0291738.s003.tif]
